# The HLA class-II immunopeptidomes of AAV capsids proteins

**DOI:** 10.3389/fimmu.2022.1067399

**Published:** 2022-12-20

**Authors:** Carlos A. Brito-Sierra, Megan B. Lannan, Robert W. Siegel, Laurent P. Malherbe

**Affiliations:** Lilly Research Laboratories, Eli Lilly and Company, Indianapolis, IN, United States

**Keywords:** AAV (Adeno-associated virus), immunogenicity, immunopeptidome, gene therapy, risk assessment, CD4+, HLA

## Abstract

**Introduction:**

Gene therapies are using Adeno-associated viruses (AAVs) as vectors, but immune responses against the capsids pose challenges to their efficiency and safety. Helper T cell recognition of capsid-derived peptides bound to human leukocyte antigen (HLA) class II molecules is an essential step in the AAV-specific adaptive immunity.

**Methods:**

Using MHC-associated peptide proteomics, we identified the HLA-DR and HLA-DQ immunopeptidomes of the capsid proteins of three different AAV serotypes (AAV2, AAV6, and AAV9) from a panel of healthy donors selected to represent a majority of allele usage.

**Results:**

The identified sequences span the capsids of all serotypes, with AAV2 having the highest peptide count. For all the serotypes, multiple promiscuous peptides were identified and displayed by both HLA-DR and -DQ. However, despite high sequence homology, there were few identical peptides among AAV2, AAV6, and AAV9 immunopeptidomes, and none were promiscuous.

**Discussion:**

Results from this work represent a comprehensive immunopeptidomics research of potential CD4+ T cell epitopes and provide the basis for immunosurveillance efforts for safer and more efficient AAV-based gene therapies.

## 1 Introduction

Adeno-associated viruses (AAVs) are widely investigated as delivery platforms in gene therapies. AAVs have multiple attributes that make them ideal vectors: they are non-integrative, have high transduction efficiency, sustained expression of transgene and ability to transduce both dividing and non-dividing cells (1–4). At the time of manuscript preparation, there are 288 clinical trials using AAVs and two FDA-approved gene therapies: LUXTURNA^®^ and ZOLGENSMA^®^, for treating inherited retinal disease and spinal muscular atrophy, respectively (5). Despite their multiple advantages, clinical data have shown that the efficiency and safety of AAV-based gene therapies can be affected by the immune responses to the virus itself as well as the transgene.

The capsid is the only viral protein present in the AAV particles used in gene therapy. The immune system recognizes viral capsids and elicits adaptive immunity to AAVs in the form of humoral and cellular responses. Pre-existing immunity is a major challenge in gene therapy, given that a large fraction of the human population has been exposed to AAVs at some point in their lives. For example, between 30 and 60% of humans have neutralizing antibodies against AAV serotypes 1 to 9 (6). If an AAV therapeutic is detected by a neutralizing antibody, the viral particle might be degraded without delivering the therapeutic gene. Another challenge is the presence of memory T cells. Different clinical trials have documented robust cellular immune responses developed by patients upon treatment with AAV (7, 8). Circulating capsid-specific CD4+ and CD8+ T cells are common among adults (9) and pose a risk when re-dosing because activated CD8+ cells can lyse transduced cells and lead to a reduction of the therapeutic gene (8–10).

Activation of CD4+ T cells plays a central role in the initiation of the adaptive cellular and humoral immune responses to AAVs. AAV-specific CD4+ T cells recognize, through their T cell receptors, capsid-derived peptides presented by HLA class-II molecules on the surface of antigen-presenting cells. While a number of studies have identified CD8+ T cell epitopes in AAV2 capsid (11–14), very little is known about the T cell epitopes recognized by AAV-specific CD4+ T cells (15). MHC-associated peptide proteomics (MAPPs) is a recent technology that gives access to the HLA class II immunopeptidome, defined as the naturally displayed peptides by HLA class II (DR, DQ and DP) molecules on the surface of human dendritic cells. Recent studies have leveraged MAPPs to identify immunopeptidomes of different viruses, including SARS-CoV-2, influenza, and HIV (16–21) as well as immunogenicity risk assessment of preclinical molecules in the industry (22). However, despite the importance of AAVs in gene therapies, no previous MAPPs studies have been performed on AAV capsids.

In the present study, we aimed to identify the HLA class-II immunopeptidome of three AAVs frequently used in gene therapy: AAV2, AAV6, and AAV9. Monocyte-derived dendritic cells were isolated from ten donors and independently pulsed with the capsid protein of three AAV serotypes. For each serotype and donor, HLA class-II peptides were immunoprecipitated from two major class-II loci: HLA-DR and -DQ and identified using liquid chromatography and nanoelectrospray ionization tandem mass spectrometry. We observed prominent and differential peptide presentation among the three serotypes, with higher peptide numbers on HLA-DR. Peptides derived from AAV6 capsid were observed in lower numbers compared to AAV2 and AAV9. We identified highly promiscuous peptides displayed among multiple donors and determined that those are also shared between the HLA-DR and -DQ immunopeptidomes. Finally, we identified a group of conserved peptides shared among the three serotypes that show low promiscuity and are restricted to a smaller subset of donors. These results could set the basis for immunogenicity monitoring of AAV capsids in clinical trials and could be used to develop less immunogenic and more effective gene therapies.

## 2 Materials and methods

### 2.1 Experimental model and subject details

Buffy coats obtained with ethical approval from healthy donors who provided written consent were purchased from American Red Cross. Peripheral blood mononuclear cells (PBMCs) were isolated from the buffy coats *via* density gradient centrifugation following institutional safety guidelines. CD14+ cells were isolated from PBMCs *via* positive selection with magnetic beads and cultured at 37°C in 5% CO_2_ on 6-well culture plates in RPMI media supplemented with GM-CSF and IL-4.

### 2.2 Isolation and differentiation of monocyte derived dendritic cells

Protocol was adapted as described (23) with the following modifications. Buffy coats were diluted 1:1 in PBS, then transferred to a Ficoll-Pacque density gradient medium (GE HealthCare #17544203) in SepMate tubes (Stem Cell Technologies #86450). After 10 minutes of centrifugation at 700 g at 20°C three layers were formed: a plasma layer, the PBMC interphase and the layer of Ficoll-Paque. Two thirds of the plasma layer was aspirated off and the PBMC layer was transferred to another tube and centrifuged for 10 minutes at 700 g at 20°C. After centrifugation, the supernatant was discarded, and the pellet was resuspended in 8 mL of chilled autoMACS^®^ Running Buffer (Miltenyi Biotec #130-091-221). To enrich for CD14+ cells, 1.5 mL of CD14 microbeads (Miltenyi Biotec # 130-050-201 was added to each cell suspension and incubated for 20 minutes at 4°C Next, cells were washed in 40 mL of chilled autoMACS^®^ running buffer and centrifuged at 300 xg for 10 minutes at 4°C. After that, cell pellets were resuspended in 6 mL of the same buffer and loaded on the AutoMACS instrument for positive selection. 300 µl of the CD14 depleted PBMCs were sent to CD Genomics (https://www.cd-genomics.com/) for HLA typing. After separation, count and viability of CD14+ PBMCs were measured on the Countess II instrument (Invitrogen AMQAX1000) using trypan blue (Invitrogen #T10282). Cells were resuspended at a density of 1 x 10^6^ cells/mL in RPMI 1640 + Glutamine medium (Gibco #11875-093) supplemented with 5% serum replacement (Thermo Fisher Scientific #A2596101), 5 mM HEPES (Gibco #15630-080), 1% of MEM nonessential amino acids (GIBCO #11140-050), 100 U/mL Penicillin/Streptomycin (Hyclone #SV30010), 1 mM Sodium Pyruvate (Gibco # 11360070), 50 µM β-mercaptoethanol (Fisher chemical #O3446I-100), and 3.5% of DMEM high glucose (Gibco #31053-028). In addition, to stimulate differentiation into monocyte-derived dendritic cells, the medium was also supplemented with 40 ng/mL of granulocyte-colony stimulating factor (Human GM-CSF; Sargramostim, Sanofi-Aventis, NDC #0024-5843-05) and 20 ng/mL of IL-4 (R&D Systems, #204-IL). Finally, 5 mL of cells suspension were seeded per well in 6-well plates (Corning #353046) and incubated for four days at 37°C in an atmosphere of 5% CO_2_.

### 2.3 Dosing, maturation and harvesting of moDCs

On day 4 after isolation, moDCs were loaded with the VP1 capsid protein. The VP1 protein was chosen because it contains all the sequences present in the AAV capsid. VP2 and VP3 are shorter splice variants of VP1 and are therefore contained in the protein used for dosing. Briefly, 2.5 mL of the media was aspirated off from each well and the corresponding amount of protein was added. For the titration assays using AAV2-VP1 protein, the protein amount varied between 12 to 800 µg per donor. In contrast, the experiments addressing the HLA-DR and HLA-DQ immunopeptidome of AAV2, AAV6 and AAV9 VP1 capsid proteins, the dosing was made at 200 µg per donor. Cells were incubated at 37°C with the test article for 6 hours. Then, 2.5 mL of complete RPMI medium supplemented as described above was added to the cells along with lipopolysaccharide (LPS, at a final concentration of 1 µg/mL; Sigma-Aldrich #L5886) to induce maturation. After 24 hours, cells were harvested with 0.5 mL of RIPA lysis and extraction buffer (Thermo Fisher #89900), containing 1:1000 of 10 units/µL DNase (Roche # 04716728001) and 1 tablet of EDTA free protease inhibitor cocktail (Roche #11836170001) per every 10 mL of lysis buffer. Two wells containing the same test article and/or concentration were pooled together for a final volume of 1 mL and 200 µg of VP1 capsid protein. The samples were stored at -80°C until further processing.

### 2.4 HLA class-II peptide isolation

Immunoprecipitation of HLA class-II peptides was performed using an Agilent AssayMap robot. Briefly, 100 µg of biotinylated anti-HLA-DR (L243, produced in house), anti-HLA-DQ (SVP-L3, produced in house) or anti-pan HLA class II (Tu39 produced in house and used in this study only for the titration assay pertaining to **Figure S2**) were immobilized on streptavidin cartridges (Agilent, G5496-60010) by passing over the cartridge at 5 µL/minute and washing three times with PBS. Simultaneously, cell lysates were thawed, passed over a 0.2 µm hydrophilic filter plate (Analytical Sales & Services #96432-10) and loaded in a 96 well polypropylene plate (Thermo Scientific #AB1127). After this, the lysate was passed over the antibody-bound affinity cartridges at 5 µL/minute at room temperature (approximately 200 minutes total). Then, cartridges were washed twice with 50 mL of 100 mM ammonium acetate and once with 50 µL water at 25 µL/minute. HLA:peptide complexes bound to the specific antibodies were eluted from the cartridges with 50 µL of 5% acetic acid with 0.1% TFA at 2 µL/minute into a 96 well polypropylene PCR plate (Abgene #AB2800). Eluted peptides were passed over 10K MWCO spin filters (MilliporeSigma #MRCPRT010) equilibrated with 1mg/mL BSA (Sigma #05470), 100 µg/mL angiotensin-I and washed with 5% acetic acid. 20 µL of the filtered samples were loaded in a 96-well polypropylene PCR plate for mass spectrometry.

### 2.5 Liquid chromatography – mass spectrometry analysis of HLA class-II derived peptides

A Thermo easy-nLC 1200 system coupled to a Q-Exactive (HFX) orbitrap mass spectrometer (Thermo Scientific) was used to analyze the eluted peptides. Separation was performed with a 75 µm x 150 mm EASY-Spray HPLC column (Thermo Scientific #ES900) coupled to a standard EASY-Spray source with an electrospray potential of 1.9 kV. The solvents used were 0.1% formic acid in water (buffer A) and 0.1% formic acid in 80% acetonitrile (buffer B). A 65 minute gradient was performed using a flow rate of 250 nL/minute as follows: 60 minutes at 2-55% of B, followed by 1 minute of 55-95% of B and finally holding at 95% of B for 4.5 minutes. The Q-Exactive was run with a full scan of 120,000 resolution in the orbitrap followed by a top 20 data dependent MS/MS cycle comprised of orbitrap scans where +2, +3 and +4 ions were fragmented with HCD (CE of 15 and 25).

### 2.6 LC-MS data analysis

Raw files were analyzed using the Lilly proteomics pipeline (24). Briefly, RAW files were processed with the X! Tandem version 2017, OMSSA version 2.1.7 and ProteinPilot. A database was used consisting of the AAV2, AAV6 and AAV9 VP1 capsid protein sequences and 2134 common human and bovine proteins identified from HLA-II bound peptides seen from Raji cells, DCs and bovine proteins from the cell media. No enzyme search was set while the maximum peptide length was 30, and a 10 ppm tolerance for parent ions and 0.5 m/z tolerance of fragment ions. The potential modifications searched for included cysteine disulfides, mercaptoethanolation; mono, di, and tri oxidation; and cysteinylation; deamidation of glutamine and asparagine; methionine oxidation; tryptophan oxidation, dioxidation, oxidation to kynurenin. False positives were assessed by running the searches against a reverse version of the database and estimating false positive recovery rates. The results from X! Tandem, OMSSA and ProteinPilot were pooled and rescored. Peptides with false discovery rates (q-values) <0.20 were assigned to the smallest group of proteins that account for all identified peptides. If a particular spectra was assigned to different peptide sequences from multiple search engines, the pipeline only reports the identification with the highest scoring match. HCD spectra included b- and y- ions.

### 2.7 Quantification and statistical analysis

Data manipulation was performed in KNIME 3.3, excel, graphpad and funrich. The analysis in KNIME included: merge of all donor results, filtering for peptides specific to the VP1 capsid, filtering for peptides of 9-25 amino acid residues, manual review of ms/ms spectra for identification of at least 4 continuous fragment ions, alignment to the corresponding VP1 capsid protein and creation of an excel file with the alignments. Peptide clusters were identified using the IEDB clustering analysis tool (25), selecting the minimum sequence identity threshold at 70% and choosing the clustering method “all the connected peptides in a cluster”. Peptides associated with the donor and the HLA typing were used for identification of cores and MHC molecule using MHCMotifDecon – 1.0 (26) and the Gibbs cluster 2.0 server (27). Bar graphs, heatmaps and pie charts were made in GraphPad Prism 9 and the Venn Diagrams were created in FunRich 3.1.3 (28).

## 3 Results

### 3.1 AAV capsid-derived peptides are prominently presented by both HLA DR and DQ

To characterize the HLA class II immunopeptidome of AAVs, we dosed monocyte-derived dendritic cells (moDCs) with the VP1 capsid protein of AAV2, AAV6 and AAV9. The VP1 protein was chosen because it contains all the capsid sequences present in an AAV particle. The full-length sequence of VP1 was retrieved from GenBank (AAV2: YP_680426.1, AAV6: AAB95450.1and AAV9: AAS99264.1), expressed in BL21(DE3) cells and purified with HisPur Ni-NTA and dialysis (**Figure S1**). To determine the optimal amount of VP1 capsid protein needed to dose moDCs, we performed a titration assay. moDCs derived from two donors were dosed at AAV2-VP1 amounts ranging from 12 µg to 800 µg and the total HLA-II peptides were isolated with a pan-HLA class II antibody and characterized with the MAPPs method (see STAR Methods for details). We observed capsid specific peptides among all the concentrations tested (**Figures S2A-C**). Peptide and cluster counts were dose-dependent and peaked at 200-400 µg (**Figures S2D-H**). At the highest dose (800 µg) we observed a reduction in the peptide count. Since there was no difference in peptide counts between moDCs dosed with 200 or 400 µg, 200 µg was selected for further studies as the optimal amount of capsid dosage of moDCs.

**Figure 1:**
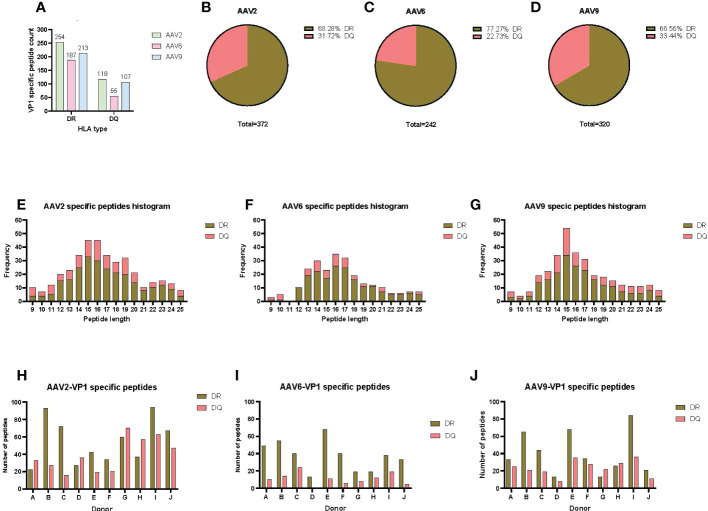
AAV serotypes are prominently presented by both HLA DR and DQ receptors. **(A)** Bar graph of the total AAV VP1 unique capsid peptide count per serotype and HLA type. **(B–D)** Pie charts representing the proportion of DR vs DQ peptides identified for AAV2 **(B)**, AAV6 **(C)** and AAV9 **(D)**. **(E–G)** Histograms representing the distribution of the identified HLA class-II peptide lengths from the VP1 capsid of AAV2 **(E)**, AAV6 **(F)** and AAV9 **(G)**. **(H–J)** Bar graphs showing total number of VP1 capsid protein peptides identified per donor for AAV2 **(H)**, AAV6 **(I)** and AAV9 **(J)**. The donor identifier is listed on the X-axis.

To have a comprehensive understanding of the HLA class-II immunopeptidome of AAV2, AAV6 and AAV9, we sought to identify the peptides presented on both HLA-DR and HLA-DQ. We first isolated moDCs from PBMCs of ten donors which accounted for a DRB1 and DQB1 frequency in the US population of 61.6% and 93.4%, respectively (**Table 1**). The HLA class II molecules were immunoprecipitated with specific antibodies for HLA-DR or HLA-DQ. Bound peptides were eluted, analyzed by capillary HPLC on an orbitrap mass spectrometer, and identified using three proteomics search engines. Large immunopeptidome datasets were further analyzed using deconvolution and clustering tools available online. The entire dataset of peptides, predicted binding cores, most probable HLA-II allele as well as alignment with the protein sequence, are shown in **Table S1**. For HLA-DR, a total of 254, 187 and 213 unique peptides were identified for AAV2, AAV6 and AAV9, respectively. For HLA-DQ the peptide counts were 3-4 fold lower, with a total of 118, 55 and 107 unique peptides for AAV2, AAV6 and AAV9, respectively (**Figures 1A-D**); **Table S1**). The length of the AAV capsid peptides eluted from HLA-DR and HLA-DQ varied from 9 to 25 aa with a mean between 15 and 16 for the three serotypes reflecting a classical HLA class-II peptide length distribution (**Figures 1E-G**). The entire immunopeptidome of the donors tested also had a similar peptide length distribution (**Figure S4**). The number of unique peptides eluted from HLA-DR or HLA-DQ was variable among donors and serotypes. The average number of unique peptides displayed per donor by HLA-DR and DQ was higher for AAV2 (55 and 39) than for AAV6 (37 and 11) or AAV9 (40 and 23) (**Figures 1H-J**).

**Table 1:** Donor HLA typing and frequencies in the US population.

Donor ID	DRB1	DRB345	DQA1	DQB1	USA DRB1 Population Frequency*	USA DQB1 Population Frequency*
A	07:01, 15:01	01:03,01:01	01:02,02:01	03:03,06:02	22.8%	17.0%
B	04:01, 15:01	01:03,01:01	01:02,03:01	03:02,06:02	16.9%	23.1%
C	04:01, 0701	01:01,01:03	02:01,03:03	02:02,03:01	18.1%	28.3%
D	07:01, 07:01	01:01,01:03	02:01,02:01	02:02,03:03	12.0%	14.0%
E	11:01,15:01	02:02,01:01	01:02,05:05	03:01,06:02	16.4%	31.3%
F	03:01, 14:54	01:01,02:02	01:04,05:01	02:01,05:03	12.5%	23.2%
G	03:01, 15:01	01:01,01:01	01:02,05:01	02:01,06:02	21.2%	33.9%
H	07:01, 13:02	03:01,01:03	01:02,02:01	03:03,06:04	16.2%	6.8%
I	04:07, 12:01	02:02,01:03	03:03,05:05	03:01,03:01	3.6%	18.3%
J	01:01, 03:01	01:01	01:01,0501	02:01,0501	17.1%	32.8%
				Total	61.6%	93.4%

*HLA haplotype frequency data was retrieved from the National Marrow Donor Program for DRB1 (https://bioinformatics.bethematchclinical.org/workarea/downloadasset.aspx?id=6398) and DQB1 (https://bioinformatics.bethematchclinical.org/workarea/downloadasset.aspx?id=6396).

### 3.2 HLA-II DR immunopeptidomes of AAVs

**Figure 2:**
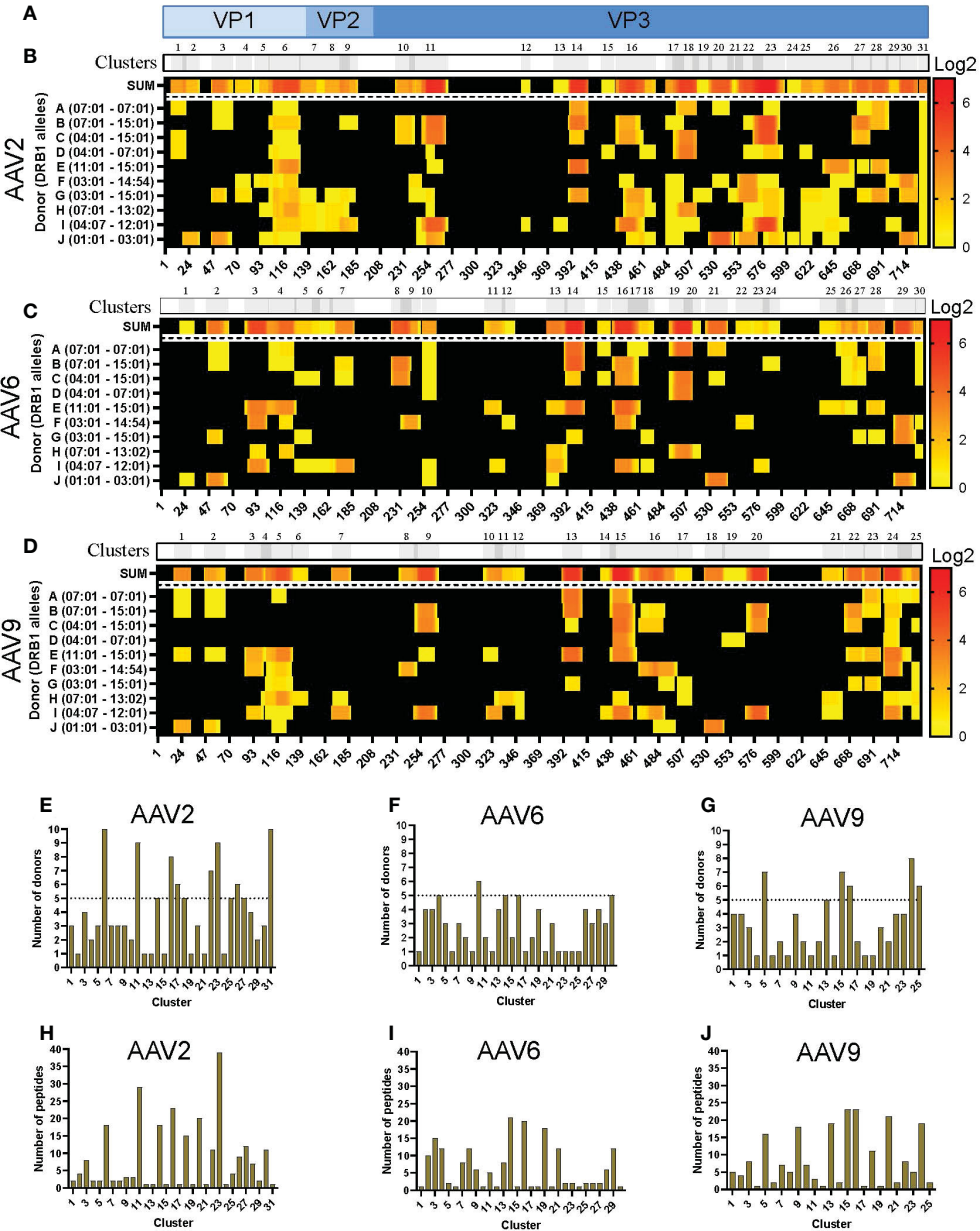
Differential levels of HLA-DR display among AAV serotypes. **(A)** Diagram representation of the full-length sequence of VP1, highlighting the regions that correspond to the VP1, VP2 and VP3 regions. **(B-D)** MAPPs peptide representation of AAV2 **(B)**, AAV6 **(C)** and AAV9 **(D)**. Aligned HLA-II peptides are displayed as heatmaps where: the first row (in grey) represents the peptide clusters, identified by IEDB Epitope Cluster Analysis Tool 1.0. Overlapping sections of peptide clusters are shown as darker grey; the second row, shown as “SUM,” represents peptides summed where each amino acid is shown as the intensity relative to the number of times it was seen on the identified peptide sequences; rows 3 to 12 represent each of the donors and their DRB1 alleles. Peptide intensity in SUM and the donor rows are shown in log2 scale, where black represents no peptide presentation and red the most presentation of peptides in a particular region. At the bottom of each heatmap, the amino acid residue position is indicated. **(E–G)** Bar graphs showing the number of donors presenting at least one peptide from each cluster AAV2 **(E)**, AAV6 **(F)** and AAV9 **(G)**. The cluster numbering as in B-D is represented on the X-axis and the number of donors is shown in the Y-axis. The dotted line represents the cutoff for “public” clusters. **(H–J)** Bar graph representing the number of peptides per cluster for AAV2 **(H)**, AAV6 **(I)** and AAV9 **(J)**. Clusters are denoted in the text by the serotype + HLA + cluster number, e.g. AAV2.DR.23.

The sequences of the peptides eluted from HLA-DR were aligned with the capsid protein of the corresponding serotype and displayed as a heatmap for relative peptide abundance visualization (**Figures 2A–D**). We found HLA-DR peptides covering 76, 59 and 63% of the capsid protein of AAV2, AAV6 and AAV9, respectively (**Figure 2**; **Figure S3A**). In order to have a broad perspective of the displayed regions along the capsid protein, we performed a clustering analysis for each serotype using the IEDB Epitope Cluster Analysis Tool 1.0 software. A cluster was defined as a family of related peptides that share at least 70% identity with at least one member of the cluster. Clusters with two or more unique peptides were designed as “nested,” versus the ones with a single peptide which are called “singletons” (**Table S2**). We identified 31, 30 and 25 clusters for AAV2, AAV6 and AAV9, respectively (**Figures 2B-D**; **Table S2**). Notably, despite multiple peptide clusters along the capsid protein, we observed several regions with no peptide display that spanned long continuous sections of the protein with identical sequence among the three AAV serotypes.

In addition, we denoted clusters presented by six or more donors as “public.” As a result, the number of public clusters varied among the serotypes. AAV2 displayed the highest number of public clusters (eight); 2 of them (AAV2.DR.6 and AAV2.DR.31) were observed in all donors sampled in this study (**Figure 2E**). AAV9 displayed 5 public clusters (**Figure 2G**). In contrast, AAV6 only displayed 1 public cluster (**Figure 2F**). Moreover, the number of peptides also varied among clusters, and as expected, nested clusters showed the highest numbers (**Figures 2H–J**). AAV2 and AAV9 had in average 8 peptides per cluster, whereas AAV6 only 6 peptides per cluster (**Table S2**).

### 3.3 HLA-II DQ immunopeptidomes of AAVs

**Figure 3:**
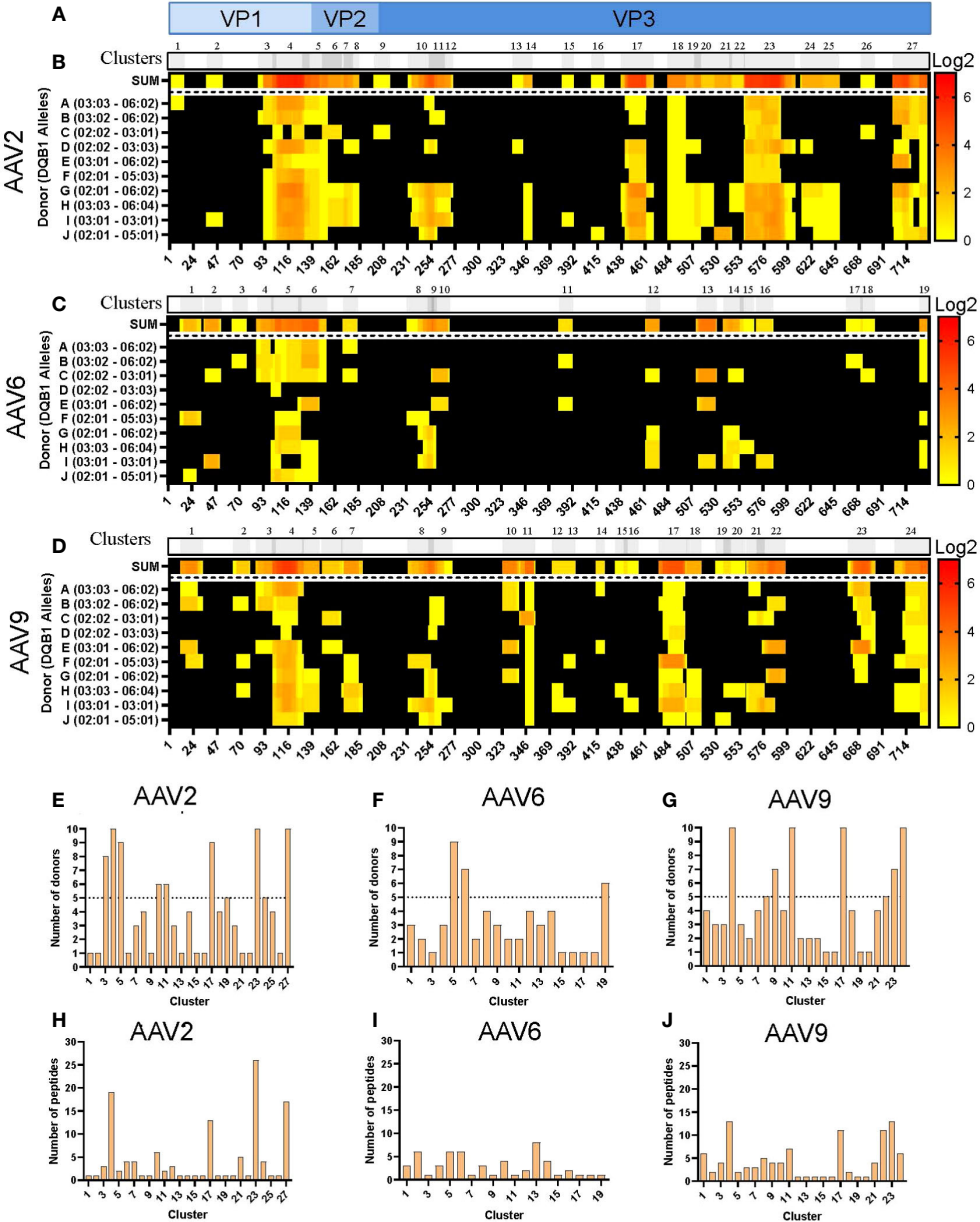
Differential levels of HLA-DQ display among AAV serotypes. **(A)** Diagram representation of the full-length sequence of VP1, highlighting the regions that correspond to the VP1, VP2 and VP3 regions. **(B–D)** MAPPs peptide representation of AAV2 **(B)**, AAV6 **(C)** and AAV9 **(D)**. Aligned HLA-II peptides are displayed as heatmaps where: the first row (in grey) represents the peptide clusters, identified by IEDB Epitope Cluster Analysis Tool 1.0. Overlapping sections of peptide clusters are shown as darker grey; the second row, shown as “SUM,” represents peptides summed where each amino acid is shown as the intensity relative to the number of times it was seen on the identified peptide sequences; rows 3 to 12 represent each of the donors and their DQB1 alleles. Peptide intensity in SUM and the donor rows are shown in log2 scale, where black represents no peptide presentation and red the most presentation of peptides in a particular region. At the bottom of each heatmap, the amino acid residue position is indicated. **(E–G)** Bar graphs showing the number of donors presenting at least one peptide from each cluster AAV2 **(E)**, AAV6 **(F)** and AAV9 **(G)**. The cluster numbering as in B-D is represented on the X-axis and the number of donors is shown in the Y-axis. The dotted lines represent the cutoff for “public” clusters. **(H–J)** Bar graph representing the number of peptides per cluster for AAV2 **(H)**, AAV6 **(I)** and AAV9 **(J)**. Clusters are denoted in the text by the serotype + HLA + cluster number, e.g. AAV2.DQ.23.

Analogous to what was observed with the HLA-DR peptides, the levels of HLA-DQ peptide presentation varied among serotypes. HLA-DQ peptides covered 65, 42 and 60% of the capsid protein of AAV2, AAV6 and AAV9, respectively (**Figure S3A**). The clustering analyses resulted in 27, 19 and 24 clusters along the VP1 capsid proteins (**Figures 3A-D**; **Table S2**). Despite clusters being distributed along the entire capsid, we also observed that the three serotypes contain long consecutive sequences that do not have peptide display on HLA-DQ (**Figures 3B-D**). Furthermore, similar to the HLA-DR clusters, the number of public clusters in the HLA-II DQ immunopeptidomes was higher for both AAV2 and AAV9 than for AAV6: 8, 6 and 3 public clusters, respectively. In addition, eight clusters were observed in all the donors, four of AAV2 and four of AAV9: AAV2.DQ.4, AAV2.DQ.18, AAV2.DQ.23, AAV2.DQ.27, AAV9.DQ.4, AAV9.DQ.11, AAV9.DQ.17 and AAV9.DQ.24 (**Figures 3E-G**; **Table S2**).

The number of peptides also varied among clusters and serotypes, with an overall peptide count higher for AAV2 and AAV9 than for AAV6 (**Figures 3H-J**). Both AAV2 and AAV9 had four clusters with more than 10 unique peptides (**Table S2**). In contrast, the cluster with the most peptide diversity of AAV6 only displayed 8 peptides (**Table S2**). Finally, we observed that in the three serotypes, there was abundant peptide display at the VP1-VP2 intersection, which corresponds to the phospholipase A2 (PLA_2_), a domain required for endosomal escape and enhancement of infectivity (29). Combined, the cluster analyses highlight regions along the capsid protein with higher HLA peptide diversity and display among the donors tested.

### 3.4 Promiscuous peptides are shared between HLA-DR and HLA-DQ

Immunodominant epitopes in proteins and pathogens are frequently promiscuous HLA class II binders (30–33). To identify promiscuous AAV epitopes (peptides observed in six or more donors in either HLA-DR or -DQ immunopeptidome), we analyzed the prevalence of individual peptides from HLA -DR and -DQ immunopeptidomes among the donors tested. We identified 23 promiscuous AAV2 peptides along eleven distinct regions of the capsid protein (**Table 2**). These peptides varied in length, from 9 to 25 amino acid residues, and were derived from the VP1 and VP3 portions of the capsid protein. Remarkably, 19 of the 23 promiscuous AAV2 peptides presented by HLA-DR were also presented on HLA-DQ. Four peptides were eluted from HLA-DQ only (**Table 2**). The majority of these highly promiscuous peptides (20 out of 23) correspond to peptides that have been shown to induce PBMC proliferation in seropositive donors (**Table 2**) (15).

**Table 2:** Promiscuous peptides of AAV2 capsid.

Peptide number	Peptide	Location	Length	HLA	Donor count DR	Donor count DQ	Subunit	T-cell activation evidence^a^
1	KYNHADAEF	92-100	9	DR,DQ	3	8	VP1	–
2	QERLKEDTSF	101-110	10	DR,DQ	6	10	VP1	1
3	QERLKEDTSFGGNLGRAVF	101-119	19	DR,DQ	1	9	VP1	1
4	KEDTSFGGNLGRAVF	105-119	15	DR,DQ	5	9	VP1	1
5	KEDTSFGGNLGRAVFQAKKRVLEPL	105-129	25	DR,DQ	8	8	VP1	2
6	GGNLGRAVFQAKKRVLEPLGL	111-131	21	DR,DQ	2	6	VP1	2
7	FQAKKRVLEPLGL	119-131	13	DQ	0	7	VP1	2
8	QAKKRVLEPLGL	120-131	12	DR,DQ	7	10	VP1	2
9	AKKRVLEPLGL	121-131	11	DR,DQ	3	9	VP1	2
10	GLVEEPVKTAPGKKRPV	130-146	17	DR,DQ	3	7	VP1	1
11	VEEPVKTAPGKKRPVEHSPVEPD	132-154	23	DR,DQ	2	9	VP1/VP2	1
12	ALPTYNNHLY	248-257	10	DR,DQ	1	6	VP3	2
13	LYYLSRTNTPSGTTTQSRLQF	442-462	21	DR,DQ	2	7	VP3	1
14	SRTNTPSGTTTQSRLQF	446-462	17	DR,DQ	3	7	VP3	1
15	YRQQRVSKTSADNNNSEY	483-500	18	DR,DQ	6	10	VP3	1
16	VMITDEEEIRTTNPVATEQY	557-576	20	DR,DQ	2	9	VP3	2
17	MITDEEEIRTTNPVATEQY	558-576	19	DR,DQ	6	10	VP3	2
18	MITDEEEIRTTNPVATEQYGSV	558-579	22	DQ	0	6	VP3	2
19	GSVSTNLQRGNRQAA	577-591	15	DR,DQ	7	10	VP3	–
20	GSVSTNLQRGNRQAAT	577-592	16	DR,DQ	5	9	VP3	–
21	TSNYNKSVNVDFTVDT	701-716	16	DQ	0	6	VP3	1
22	SNYNKSVNVDFTVDT	702-716	15	DQ	0	6	VP3	1
23	GTRYLTRNL	727-735	9	DR,DQ	10	10	VP3	2

a Immunogenic epitopes described by other studies using synthetic overlapping peptides and activation of PBMCs (15). The numbers represent the reactive donors (out of 16) to the corresponding peptide.

For AAV6 we identified 5 promiscuous peptides along 5 regions of the capsid protein. One peptide was restricted to HLA-DR (NNHLYKQISSASTG). Three peptides were displayed on both HLA-DR and HLA-DQ but only promiscuous on HLA-DQ. And one peptide was restricted to HLA-DQ (QERLQEDTSF) (**Table 3**). Similar to AAV2, the promiscuous peptides of AAV6 also varied in length, from 9 to 25 amino acid residues and were also present along the VP1 and VP3 portions of the capsid. Finally, we identified 12 promiscuous peptides derived from the AAV9 capsid protein with varying lengths between 9 and 23 amino acid residues (**Table 4**). Four of the AAV9 promiscuous peptides have been shown in previous studies to trigger CD4 T cell responses from PBMCs of healthy donors (34). Unlike AAV2 and AAV6, we found only one AAV9 promiscuous peptide restricted to HLA-DQ (QERLKEDTSFGGNLGRAVF), the rest were promiscuous on HLA-DR only (**Table 4**).

**Table 3:** Promiscuous peptides of AAV6 capsid.

Peptide number	Peptide^b^	Location	Length	HLA	Donor count DR	Donor count DQ	Subunit
1	QERLQEDTSF	101-110	10	DQ	0	6	VP1
2	QEDTSFGGNLGRAVFQAKKRVLEPF	105-129	25	DR,DQ	3	7	VP1
3	GLVEEGAKTAPGKKRPV	130-146	17	DR,DQ	2	7	VP1/VP2
4	NNHLYKQISSASTG	253-266	14	DR	6	0	VP3
5	GTRYLTRPL	728-736	9	DR,DQ	5	6	VP3

**Table 4:** Promiscuous peptides of AAV9 capsid.

Peptide number	Peptide	Location	Length	HLA	Donor count DR	Donor count DQ	Subunit	T-cell activation evidence^a^
1	QERLKEDTSF	101-110	10	DR,DQ	1	9	VP1	–
2	QERLKEDTSFGGNLGRAVF	101-119	19	DQ	0	6	VP1	–
3	GGNLGRAVFQAKKRL	111-125	15	DR,DQ	5	9	VP1	–
4	QAKKRLLEPLGL	120-131	12	DR,DQ	3	6	VP1	–
5	TDSDYQLPY	346-354	9	DR,DQ	2	9	VP3	–
6	IDQYLYYLSKTING	440-453	14	DR	6	0	VP3	Yes
7	IDQYLYYLSKTINGSG	440-455	16	DR	7	0	VP3	Yes
8	IDQYLYYLSKTINGSGQ	440-456	17	DR	6	0	VP3	Yes
9	IDQYLYYLSKTINGSGQNQ	440-458	19	DR	6	0	VP3	Yes
10	IPGPSYRQQRVSTTVTQNNNSE	479-500	22	DR,DQ	2	7	VP3	–
11	AVNTEGVYSEPRPIGTRYLTRNL	714-736	23	DR,DQ	2	7	VP3	–
12	GTRYLTRNL	728-736	9	DR,DQ	6	9	VP3	–

a Evidence of immunogenic epitopes described by other studies using synthetic peptides and activation of PBMCs by assessment of IL-12 and IFN-γ (34).

### 3.5 Few common peptides in the HLA II immunopeptidomes of AAV2, 6, and 9

**Figure 4:**
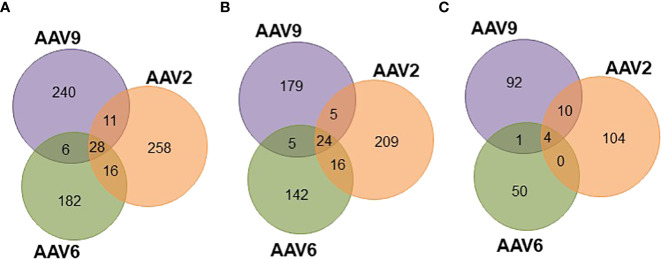
Peptides common among serotypes account for differential levels of display prevalence. Venn diagrams representing the overlap of peptides common among serotypes. **(A)** Total unique peptides identified in this study. **(B)** HLA-DR specific peptides. **(C)** HLA-DQ specific peptides.

Since the three AAV serotypes tested in this study have more than 82% sequence homology along the VP1 capsid protein (**Figures S5B, E**), a significant overlap in the HLA class II immunopeptidomes was expected. In fact, there are several regions along the capsid proteins with continuous sequence homology (e.g., between residues 274 and 326, **Figures S5A, B**) that have high cleavage probability and no biophysical properties preventing detection by mass spectrometry (data not shown). Surprisingly, out of the entire immunopeptidome dataset only 28 peptides were common among the three serotypes (**Figure 4A**; **Table S3**): 24 presented on HLA-DR (**Figure 4B**; **Figure S5C**) and 4 on HLA-DQ (**Figure 4C**; **Figure S5D**). In addition, none of these peptides were prevalent among the donors (**Figure S5F**). In fact, only one peptide (ALPTYNNHLY) was observed in more than five donors for AAV2 (six donors) but not AAV6 (three donors) or AAV9 (three donors) (**Table S3**). The rest of the peptides were restricted to four or less donors. Interestingly, all the 28 conserved peptides had been previously shown to induce CD4 T cell responses in seropositive donors (15) (**Table S3**).

## 4 Discussion

To develop safe and efficient gene therapies, it is imperative to understand the adaptive immune responses to AAVs. As a fundamental step in the adaptive immune response, antigen presenting cells display peptides in the context of HLA class II molecules to activate AAV specific CD4+ T cells. In this study, we identified the HLA class-II immunopeptidome of the capsids of three AAV serotypes currently investigated in clinical trials: AAV2, AAV6 and AAV9. The results of this study include resolution to the level of HLA loci that is carrying the peptide: either on HLA-DR or HLA-DQ. Peptide display was prominently observed among the three serotypes and the ten donors tested. AAV2 showed the highest peptide count whereas AAV6 showed the lowest among the three serotypes. Similarly, peptide promiscuity and the number of peptides displayed in both HLA-DR and HLA-DQ was lower for AAV6 than AAV2 and AAV9.

Traditionally, the studies of HLA peptidomics have focused on peptides presented on HLA-DR. This is unsurprising given that DR is the HLA class-II loci with the highest expression (35). In contrast, HLA-DQ peptides have been less characterized, but they are important for protection against JC polyoma virus (36), hepatitis B virus (37, 38) and herpes simplex virus (39). In our study, we expanded the HLA immunopeptidomics to also include the HLA-DQ peptidome. As expected, a large fraction of the HLA-DR and -DQ immunopeptidomes identified in this study were distinct. However, we observed that a number of peptides were displayed by both DR and DQ (**Figures S3B-D**). This was particularly common among the promiscuous peptides of AAV2, AAV6 and AAV9. Our results are consistent with previous studies showing that identical peptides can be eluted from both HLA-DR and -DQ (40–43).

The immunopeptidomes identified in this study contain peptides that align along the entire capsid sequences. We identified prevalent display of peptides at the VP1 and VP2 sections for all the AAV serotypes, especially on HLA-DQ. These sections of the capsid are characterized for having a highly disordered region (44) and contain the putative domains of phospholipase A2 (PLA2) (29, 45) and three nuclear localization signals (46, 47). However, peptide display from these regions might account for lower immunogenicity risk, given that the VP1:VP2:VP3 ratios correspond to 1:1:10 and are less frequently seen in AAV particles (46). Another region of particular interest is at the C-terminal of the protein, where the peptide GTRYLTRNL, seen in AAV2 and AAV9, was displayed among multiple donors. One explanation is that the terminal regions of the proteins require only one cleavage site to generate a free peptide and result in higher probability on encountering an HLA molecule. Interestingly, a peptide corresponding to this region in AAV6 was less promiscuous presumably due to Asn (N) to Pro (P) change.

In contrast, there are regions along the capsid that do not display peptides. For example, despite having high cleavage probability (NetMHCIIpan, data not shown) and no obvious biophysical properties preventing detection by mass spectrometry, we did not observe peptides in the region between residues 274 to 326, which is identical among the three serotypes and spans a large fraction of the protein. Similarly, we did not observe peptides between 217-223 and 322-338, which correspond to the pore forming amino acids of the viral capsid (48), and are also identical among serotypes. This phenomenon speaks for some type of processing or other evolutionary implications not understood. A recent study also found that regions along the SARS-CoV-2 spike protein are devoid of HLA class-II epitopes and are associated with glycosylation sites (19). However, the protein used in our assays did not have glycosylation sites because it was produced in *E. coli*. Another possibility is that these regions can be presented by HLA-DP. However, we also performed immunoprecipitations using a pan-HLA class II antibody (Tu39) which would allow the identification of HLA-DP peptides if present in sufficient quantity, and observed no peptide presentation in the same regions (**Figure S6**).

Because of the immunogenicity frequency of AAVs in gene therapies, there is a regulatory expectation to monitor T cell responses in the clinic. Currently, most clinical trials use overlapping 15-mers covering the total of the AAV capsid sequence to monitor AV-specific T cell responses. Our study identifies the most promiscuous AAV capsid peptides presented by HLA-DR and -DQ, the two major HLA class II molecules that are involved in the initiation of CD4 T cell responses. Many of these promiscuous peptides have been shown to elicit T cell responses in donors previously exposed to AAV (15). These promiscuous peptides could therefore represent an alternative to the overlapping peptide strategy to monitor AAV-specific CD4 T cell responses during clinical trial. Future studies will determine the prevalence and magnitude of the CD4 T cell responses elicited by these promiscuous peptides in exposed individuals and attempt to extend this characterization to the HLA class I immunopeptidome.

## Data availability statement

The datasets presented in this study can be found in online repositories. The names of the repository/repositories and accession number(s) can be found below: https://massive.ucsd.edu, MSV000089982.

## Author contributions

Experimental design, investigation, analysis, data curation, visualization, editing, writing – original draft: CB-S. Experimental design, data curation, writing – review & editing: ML. Supervision, analysis, writing– review & editing: RS. Supervision, analysis, writing – review & editing: LM. All authors contributed to the article and approved the submitted version.

## Supplementary material

Supplementary Table 1 Full peptides list and alignment to the protein sequence. Related to **Figures 1**–**3**.

Supplementary Table 2 Clusters identified in the study. Related to **Figures 2**, **3**.

Supplementary Table 3 Conserved peptides. Related to **Figure 4**.
