# Erratum: The HLA class-II immunopeptidomes of AAV capsids proteins

**DOI:** 10.3389/fimmu.2023.1261322

**Published:** 2023-07-27

**Authors:** Frontiers Production Office

**Affiliations:** Frontiers Media SA, Lausanne, Switzerland

**Keywords:** AAV (Adeno-associated virus), immunogenicity, immunopeptidome, gene therapy, risk assessment, CD4+, HLA

Due to a production error, an author name was incorrectly spelled as “Robert J. Siegel”. The correct spelling is “Robert W. Siegel”. The publisher apologizes for this mistake.

The original version of this article has been updated.

