# Supplementary material for: The HLA class-II immunopeptidomes of AAV capsids proteins
Source: Front Immunol. 2022 Dec 20;13:1067399. doi: 10.3389/fimmu.2022.1067399 (PMC9807805; doi:10.3389/fimmu.2022.1067399)
Supplement: Supplementary file 4 [file DataSheet_1.pdf]

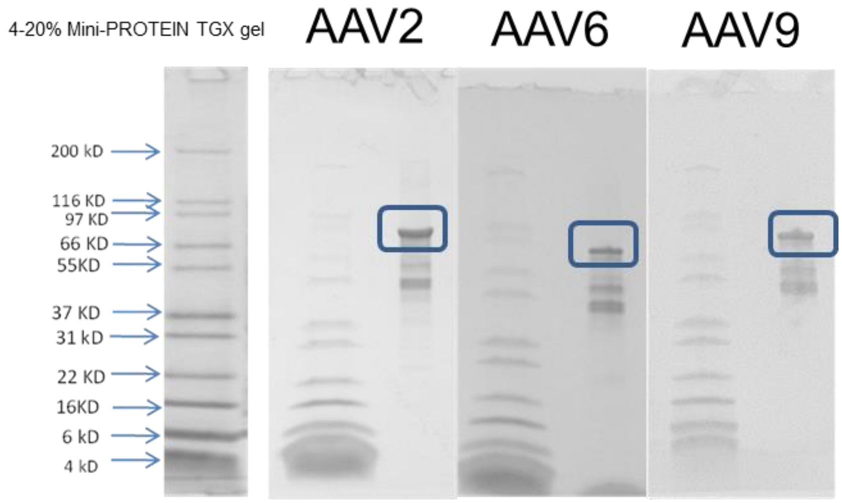

Figure S1. Purified VP1 capsid proteins.

Coomassie blue stained gel with the corresponding protein ladder showing. The VP1 bands are shown in a blue square where the sizes for VP1 are 83.3 kDa (AAV2), 82.8 kDa (AAV6) and 82.8 kDa (AAV9). Full-length capsid protein is denoted with box. Lower bands are C-terminal truncated fragments likely due to proteolytic degradation during purification using N-terminal 6x-his tag.

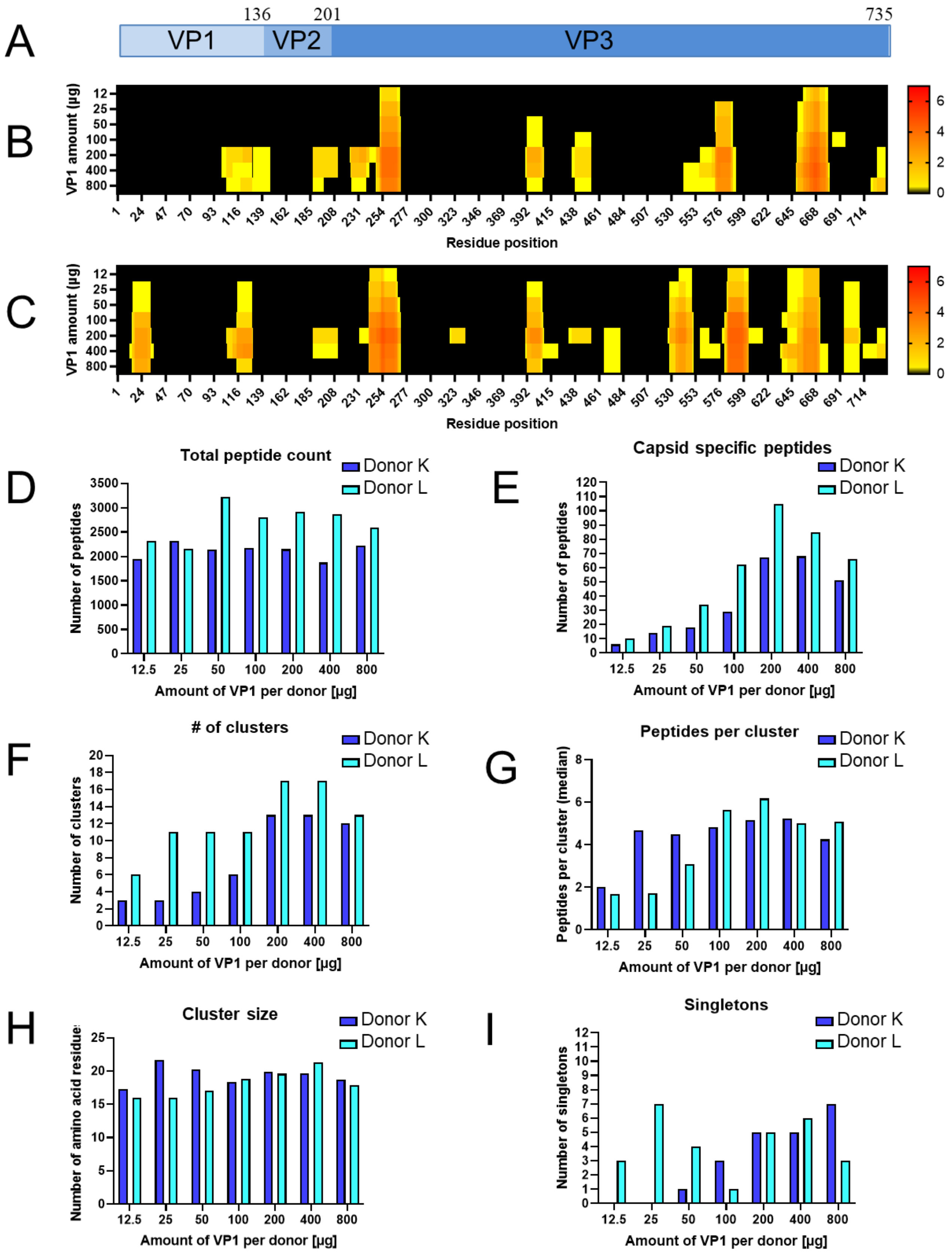

A
136
201
735
VP1
VP2
VP3
B
VP1 amount (µg)
12
25
50
100
200
400
800
Residue position
C
D
Total peptide count
Donor K
Donor L
Number of peptides
Amount of VP1 per donor [µg]
E
Capsid specific peptides
F
# of clusters
Number of clusters
G
Peptides per cluster
Peptides per cluster (median)
H
Cluster size
Number of amino acid residues
I
Singletons
Number of singletons

Figure S2. Titration of AAV2-VP1 protein in the MAPPs assay.

Seven amounts of VP1 protein were tested in two donors. The HLA class-II peptides were isolated using a pan HLA-II antibody (Tu 39), analyzed on LC-MS and proteomics search engines. (A) Diagram representation of the full-length sequence of VP1, highlighting the regions that correspond to the VP1, VP2 and VP3 regions. (B & C) Heatmaps of the peptides identified at different concentrations of VP1 from donors “K” (B) and “L” (C). The heatmaps are shown in log2 scale where black represents no peptide presentation and red the most presentation of peptides in a particular region. Each row in the heatmap is a different amount of VP1, ranging from 12 µg to 800 µg as indicated. (D) Total peptide count identified on HLA class-II. No difference was observed, among the concentrations, implying that the overall health of the cell is not affected by the different amounts of VP1 tested. (E) VP1 specific peptides at different amounts of dosed protein. 200 µg showed the greatest number of peptides. (F) Number of clusters at different amounts of dosed protein. 200 and 400 µg showed the most clusters identified. (G) Peptides per cluster identified at different amounts of VP1. 200 µg was the amount of VP1 that resulted in the most peptides per cluster. (H) Average cluster length, defined as the size of the concatenated peptides. (I) Singletons (defined as unique peptides which do not share homology above 70% to any cluster), were more common at higher concentrations of dosed VP1 capsid protein.

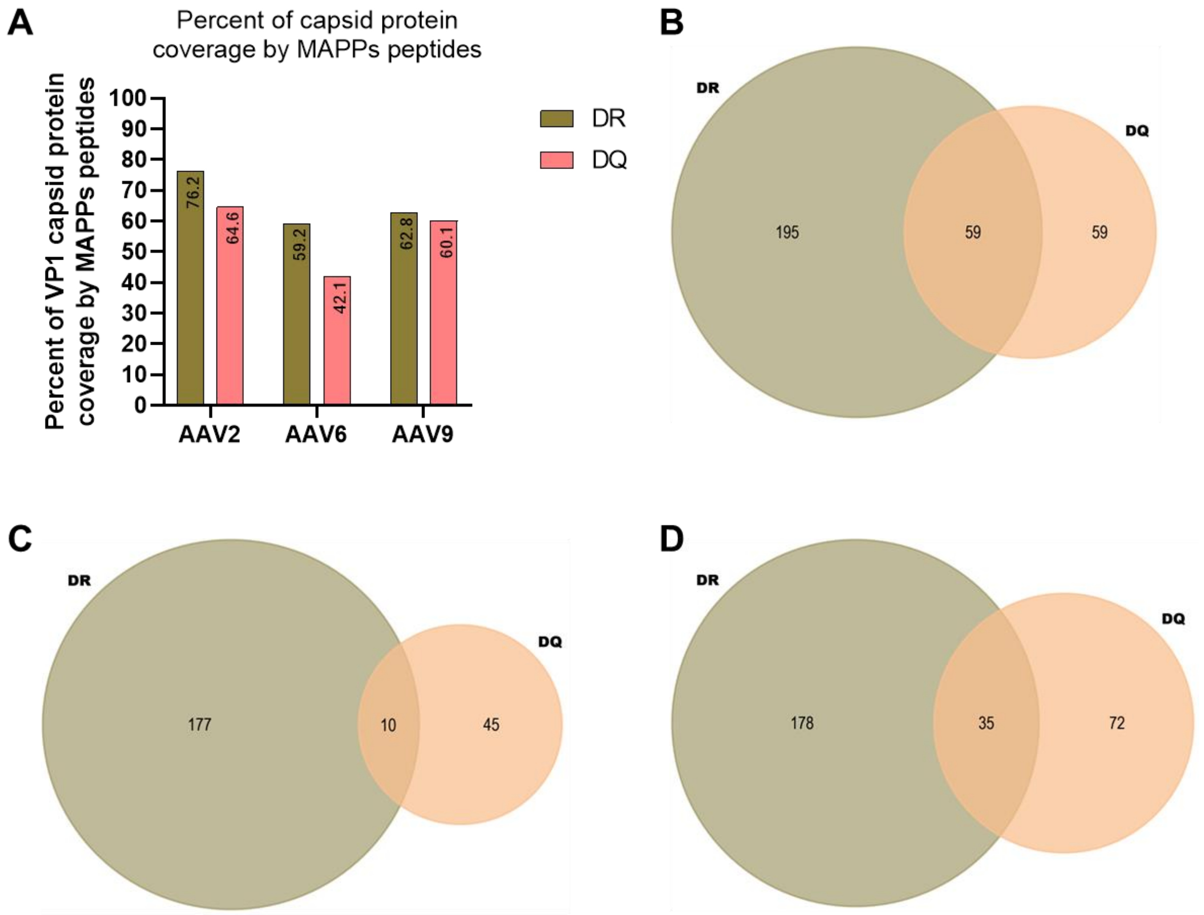

Figure S3. MAPPs peptide percentage and overlapping peptides derived from HLA-DR and HLA-DQ.

(A) Percent of the VP1 capsid protein coverage. The y-axis represents the percentage of amino acid residues within the VP1 capsid protein that were seen in MAPPs peptides relative to the total number of amino acids in the protein. (B-D) Venn Diagrams showing the number of peptides unique and overlapping between HLA-DR and HLA-DQ. (B) AAV2, (C) AAV6 and (D) AAV9.

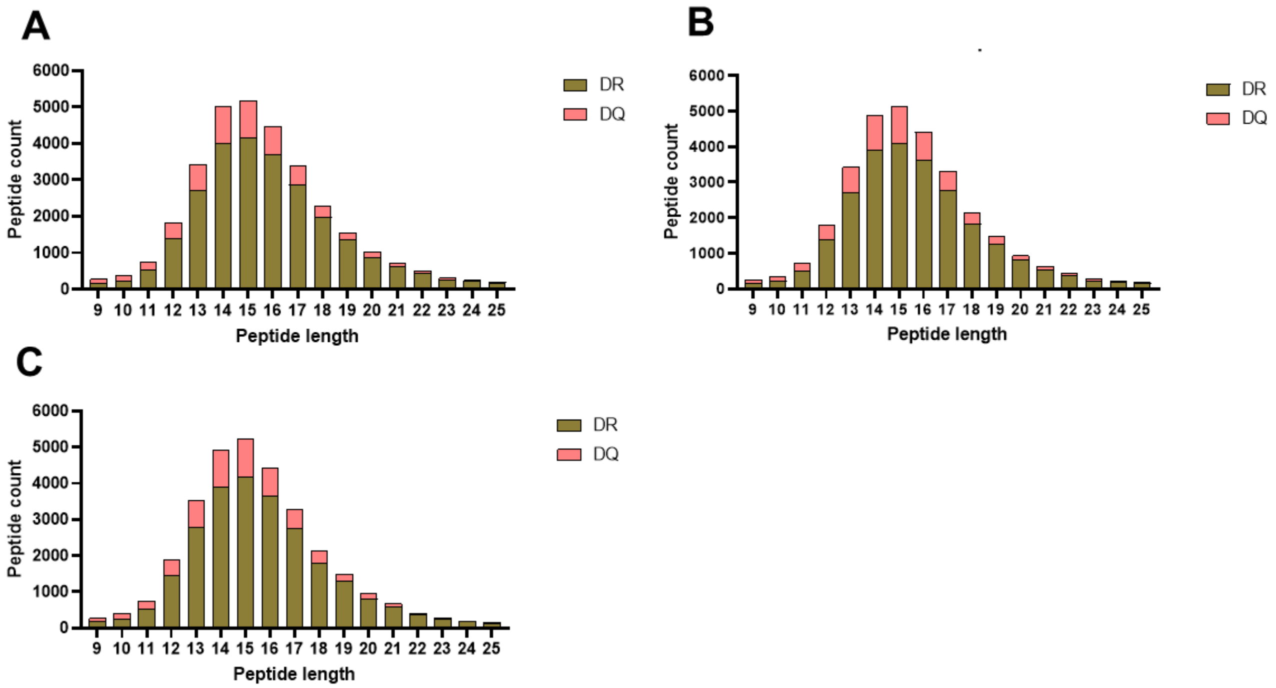

Figure S4. HLA class II peptide length distribution of the entire immunopeptidome

(A-C) Peptide size distribution observed in the entire HLA-DR and HLA-DQ immunopeptidomes of the samples treated with AAV2 (A), AAV6 (B) or AAV9 (C)

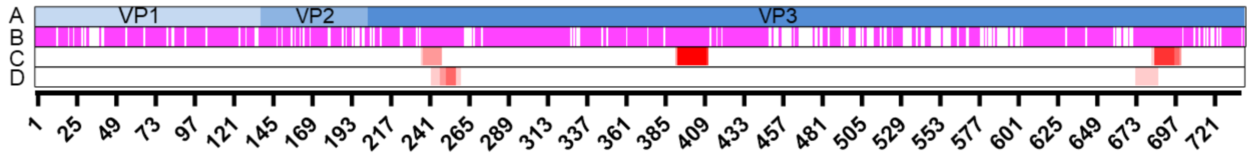

E

	AAV2	AAV6	AAV9
AAV2	100	83.4	82.02
AAV6	83.4	100	82.31
AAV9	82.02	82.31	100

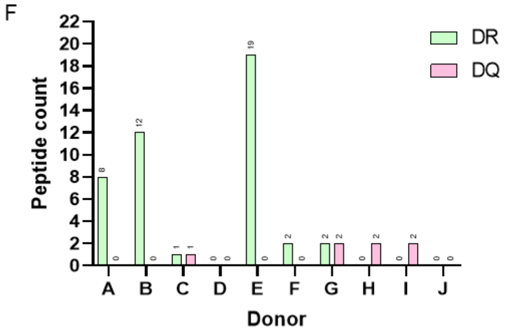

Figure S5. Conserved peptides displayed by the three AAV serotypes.

(A). Diagram representation of the full-length sequence of VP1, highlighting the regions that correspond to the VP1, VP2, and VP3 regions. (B) Conserved regions among the three serotypes. The regions colored in magenta represent matching amino acid residues among the three serotypes. (C & D) Map of peptides displayed by the three serotypes. (E) Identity percentage of the VP1 capsid protein among the serotypes. (F) Bar graph representing the number of conserved peptides counts per donor. The y-axis contains the number of peptides that are common in the three serotypes

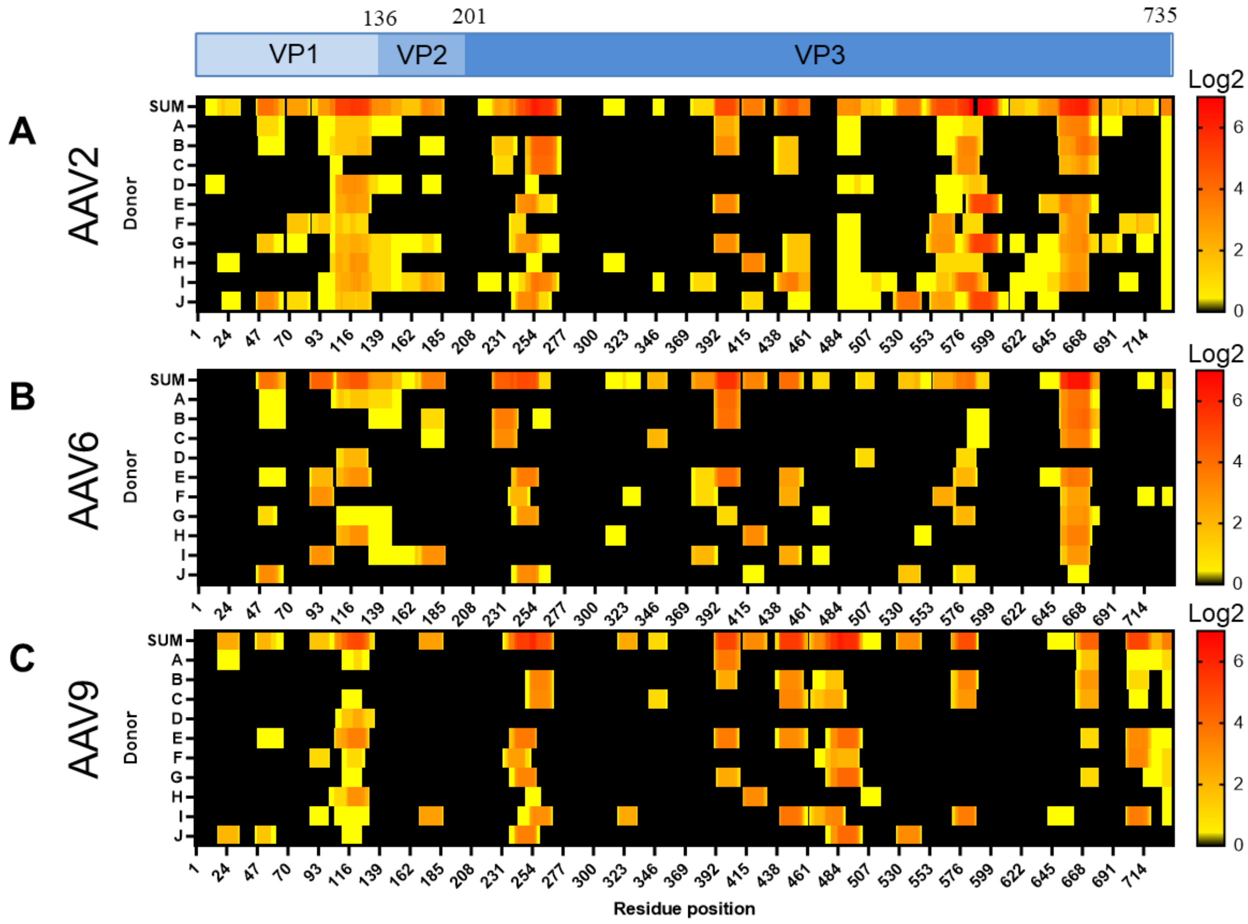

Figure S6. Pan HLA class II immunopeptidomes of AAV2, AAV6 and AAV9.

Heatmaps representing AAV-derived peptide display on HLA-II molecules. These heatmaps are showing peptides isolated using a pan-HLA class II antibody (Tu39) of moDCs dosed with AAV2 (A), AAV6 (B) or AAV9 (C).
